# Elastic Tactile Sensor Glove for Dexterous Teaching by Demonstration

**DOI:** 10.3390/s24061912

**Published:** 2024-03-16

**Authors:** Philipp Ruppel, Jianwei Zhang

**Affiliations:** Department of Informatics, Universität Hamburg, 22527 Hamburg, Germany; jianwei.zhang@uni-hamburg.de

**Keywords:** tactile sensing, sensor gloves, stretchable sensors, stretchable electronics, teaching by demonstration, imitation learning, robot learning

## Abstract

We present a thin and elastic tactile sensor glove for teaching dexterous manipulation tasks to robots through human demonstration. The entire glove, including the sensor cells, base layer, and electrical connections, is made from soft and stretchable silicone rubber, adapting to deformations under bending and contact while preserving human dexterity. We develop a glove design with five fingers and a palm sensor, revise material formulations for reduced thickness, faster processing and lower cost, adapt manufacturing processes for reduced layer thickness, and design readout electronics for improved sensitivity and battery operation. We further address integration with a multi-camera system and motion reconstruction, wireless communication, and data processing to obtain multimodal reconstructions of human manipulation skills.

## 1. Introduction

**Figure 1:**
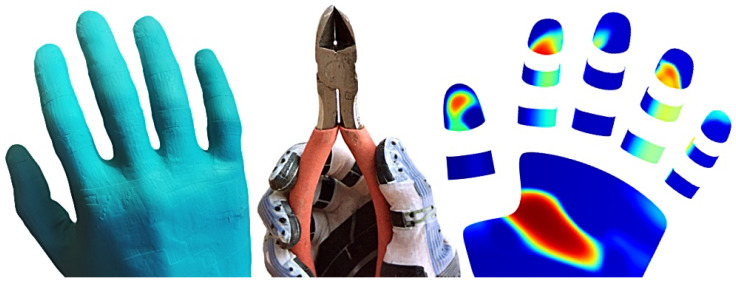
We create thin and elastic sensor gloves from elastic rubber-like materials (**left**, **center**) to capture tactile data (**right**, highest forces in red) during dexterous manipulation and tool use (**center**).

Robot teaching by human demonstration [1,2,3,4] could potentially lead to a general solution for automating work that humans currently have to perform themselves. In order to maximize the set of tasks that can be economically automated, it would be desirable to minimize the difference between working with one’s own hands and programming a robot to perform the same tasks, using physical interaction between human hands and objects or tools as a programming language. This language, however, is inherently multimodal. Humans typically manipulate objects through physical contacts. We develop sensor gloves to capture contact dynamics on human hands during dexterous manipulation (Figure 1).

The sensor gloves should be thin and elastic to avoid impeding human dexterity, the sensors should be produced in multi-curved shapes to match human physiology, and the devices should not pose health risks to human wearers. Recent scientific research into artificial sensor skin is coming increasingly closer towards fulfilling the requirements [5,6,7,8,9]. However, current sensor gloves still use traditional materials, production methods, and sensor geometries [10,11,12,13,14]. We develop a thin and elastic tactile sensor glove that covers the human hand as an artificial second skin and allows for the capture of contact dynamics during dexterous manipulation. The glove, including the sensors, electrodes, and conductors, is completely made from stretchable elastomer-based materials and uses capacitive tactile sensing. The readout electronics are contained in a wireless wrist-worn interface module, not requiring any rigid parts on the glove itself. Our main contributions over our previous work [5] are as follows:

- We develop a tactile glove with signal routing and sensors on all fingers and the palm.
- We minimize thickness through more extensive use of solvent-based processing to avoid impeding human dexterity.
- We revise our conductive silicone formulation for higher filler content yet increased stretchability and simplified curing without heating or ventilation.
- We replace lower-sensitivity closed-celled or higher-thickness in situ molded dielectrics with a thin and sensitive open-celled dielectric.
- We adapt the assembly process to reduced part thickness.
- We modify our processes to efficiently produce curved conductive trace bundles.
- We replace our readout state machine with a high-throughput pipelined architecture.
- We integrate our sensor glove with a multi-camera system and produce multimodal reconstructions of dexterous manipulation tasks.

We first design a CAD model of the glove and improve methods for partial procedural generation of the components (Section 2.1). In order to physically manufacture the glove, we develop material formulations (Section 2.3) and production techniques (Section 2.5, Section 2.6, Section 2.7 and Section 2.8). We design electronics for the wearable interface module (Section 2.9) and logic for sensor readout and data pre-processing (Section 2.10). The sensor glove is integrated with a multi-camera system and motion tracking for multimodal reconstruction (Section 2.11 and Section 2.12). We finally present the first results (Section 3) and conclusions (Section 4).

## 2. Materials and Methods

### 2.1. Glove Design

Our sensor glove design features double-curved capacitive sensor matrices wrapping around the lower, left, right, and front sides of the finger tips, U-shaped sensor strips around the bottom and sides of the middle and proximal phalanges, and an irregularly shaped multi-curved sensor matrix covering the palm and the ball of the thumb. The sensor cells are connected to a wrist-worn readout module via electrically conductive traces, which run along the top of the fingers, the back of the hand, and through a flat cable-like extension from the glove to the wrist. All sensor elements are multiplexed into a single logical readout matrix via connections on the back of the hand.

**Figure 2:**
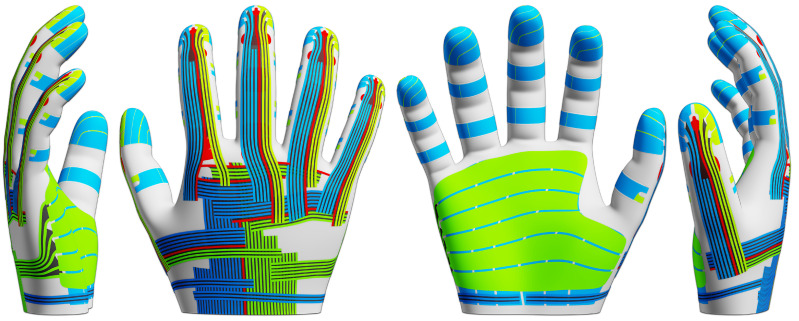
CAD model of our sensor glove. Electrodes for sensor rows and columns are shown in green and blue, spacing constraints in black, and ground electrodes in red.

We first create a CAD model of the glove in Blender [15], starting from an existing subdivision surface [16] mesh of a human hand [17]. The shape is retopologized and slightly smoothed at the nails and joints for easier casting. We then model the shapes of the electrodes, dielectric pads and interconnections and project the parts onto the hand model. Borders between neighboring electrodes are first marked via bevel weights, and after projection, we separate the electrodes by a small uniform distance using a bevel modifier, material replacement and geometry nodes. The interconnections are arranged in evenly spaced bundles, which we model as quad grids with alternating material assignments for traces and gaps. See Figure 2 for renderings of the glove model with sensor electrode shapes and interconnections. We generate a mold for casting and assembling the glove by cutting thin grooves as alignment markers around all parts into the hand model. Points for vertical connections are marked for later reference as a point set.

For mapping between tactile measurements and hand geometry, we use texture coordinate channels in Blender to assign edges of the part shapes to the corresponding row and column indices in the readout matrix. We then procedurally find the closest point on the sensor elements for each hand vertex and project the tactile sensor matrix coordinates onto the hand model. The projected tactile coordinates are combined with bone indices and skinning weights from the original hand model for integration with motion capture.

### 2.2. Sensing Principle

**Figure 3:**
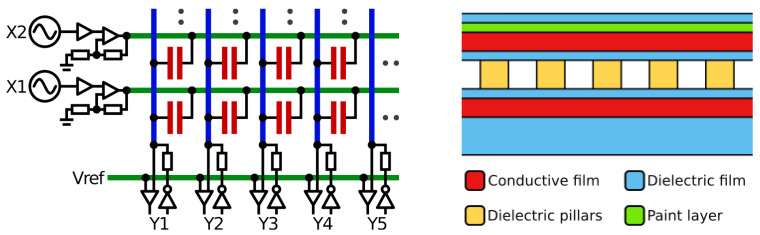
Electrical diagram (**left**) of a matrix of capacitive tactile sensors (red) with excitation lines (green), excitation drivers (X), reception lines (blue), reference voltage (Vref), and reception circuits (Y). Layer structure (**right**) of a cell on our sensor glove with a compressible pillar structure (yellow) between conductive layers (red).

Our tactile sensor cells form force-sensitive variable capacitors (Figure 3, right). A compressible dielectric pillar structure is arranged between two electrically conductive silicone films. The films are insulated with additional non-conductive silicone. The sensor cells are connected as a sensor matrix (Figure 3, left) with intersecting excitation and reception lines and capacitive tactile sensor cells at the intersections. The sensor cells are read by simultaneously applying sine waves (Figure 3, X) of different frequencies to the excitation lines and measuring the electrical current (Figure 3, Y) at the reception lines. The receivers are implemented as delta–sigma converters, which hold the reception lines at a constant voltage using input comparators and output drivers with reference resistors, tracking the currents at the output drivers required to balance the currents from the sensor cells.

### 2.3. Material Formulations

We manufacture our sensor gloves from custom addition-cure silicone formulations (see Table 1). Since we are casting the material in layers, compromises between viscosity, softness, and strength [18] or long cure times [5,19,20], which are commonly faced in the formulation of molding silicones [18,19,20], can be avoided through solvent-based processing. We start with a high molecular weight vinyl-functional methyl siloxane gum (CAS 67762-94-1, Vinyl Gum 0.074, Nedform BV, Geleen, The Netherlands) ($M_{r}=$ 650,000) for strength, softness and short cure times, and dissolve it in hexamethyldisiloxane (CAS 107-46-0, hexamethyldisiloxane, WACKER AK 0,65, silikonfabrik.de, Bad Schwartau, Germany), a volatile silicone oil, for easy processing and reduced layer thickness. We then add a hydride-functional siloxane crosslinker (CAS 69013-23-6, Crosslinker 200, Evonik Industries AG, Essen, Germany) and fillers. In the non-conductive layers, we use a hydrophobic fumed silica filler (CAS 68909-20-6, Aerosil R 812 S, Evonik Industries AG, Essen, Germany) as a reinforcing agent and a small amount of zinc oxide to increase the opacity for easier assembly. For the electrodes and conductive traces, we add carbon black as an electrically conductive filler. We choose a specialty grade with low amounts of contaminants originally intended for food contact applications (CAS 1333-86-4, Food Contact Royale Black PP802, PCBL Ltd., Profiltra, Almere, The Netherlands) to minimize cure inhibition. We also add a small amount of hydroxy-terminated PDMS (CAS 70131-67-8, polydimethylsiloxane, hydroxy terminated, M.W. 4200, VWR International GmbH, Radnor, PA, USA) for in situ filler treatment.

In order to mark parts for easier assembly and to uniformly color the glove for motion tracking with existing hand-tracking neural networks, we prepare crosslinking silicone paints by further diluting our above non-conductive formulation with additional hexamethyldisiloxane and adding pigments (Phthalocyanine (PG7, PG36, PB), Ultramarine Blue, Chromium Oxide Green - Kremer Pigmente, Aichstetten, Germany) for coloring and titanium dioxide as an opacifier.

For internal dielectric structures, which we cast using closed two-part molds and then mount between stabilizing solvent-cast layers, we also create a formulation with a lower-viscosity vinyl-functional siloxane (CAS 68083-19-2, Vinyl Dimethicone 2.000 cSt, Nedform BV, Geleen, The Netherlands), a hydride-terminated PDMS as a chain extender (Modifier 2.6, both sides Si-H end-capped, Nedform BV) and no solvent. This mixture is similar to common molding silicones but is known to not contain plasticizers or cost-saving liquid fillers that might otherwise leak out and gradually change the characteristics of the thin dielectric columns [18]. For easier demolding, we add a small amount of zinc stearate as an internal mold release agent, which should be washed off with isopropyl alcohol after curing to ensure reliable bonding with subsequent layers.

The materials can be premixed and stored for later use. We keep the solvent-based formulations in industrial Aluminium bottles (Aluminium bottles, narrow neck, ESSKA.de GmbH, Hamburg, Germany) and protocol the weights to rule out leakage and changes in solvent content. Before use, we shake the container, remove the required amount and mix with a catalyst (Karstedt’s catalyst solution with 3‰ tetravinyl tetramethyl cyclotetrasilozane, Nedform BV)) to initiate crosslinking. Tools, bottles, molds, etc., should be thoroughly cleaned (e.g., with isopropanol) and only be handled with clean tools or vinyl gloves to avoid cure inhibition. In addition to the silicones, we also prepare a release agent dispersion by stirring a small amount of zinc stearate with isopropyl alcohol.

### 2.4. Fabrication Steps

**Figure 4:**
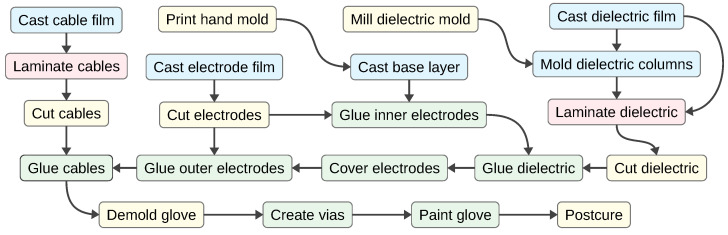
Manufacturing process for our sensor glove.

We produce our sensor glove by first casting a base layer on a 3D-printed glove mold, then casting, molding and cutting functional parts, transferring these parts onto the glove in multiple layers, and finally connecting conductors on different layers, painting the glove and post-curing the material. See Figure 4 for a schematic overview of our fabrication process and the following sections for details on the manufacturing steps.

### 2.5. Glove Molding

We print our mold model (Section 2.1) on a regular SLA printer in a black-colored resin and fill the previously generated alignment grooves (Section 2.1) with a white putty (Molto Holzkitt, Bauhaus AG, Belp, Switzerland). Excess putty can be removed using a sponge and water or isopropanol. We then apply a plastic primer (OBI Kunststoff-Grundierung Spray Transparent matt, OBI Home and Garden GmbH, Wermelskirchen, Germany)) and a clear varnish (DUPLI-COLOR 385858 CAR’S, European Aerosols GmbH, Haßmersheim, Germany). After waiting for the varnish to dry and cleaning the mold with isopropanol, we prepare a suitable amount of solvent-based dielectric silicone (Table 1) and pour it over the mold, first onto each side of each finger and then onto the palm, the sides, and the back of the hand, to create a continuous base layer. The base layer should be applied quickly enough before too much of the solvent evaporates in order to avoid discontinuities. After the material has been dispensed and the solvent has evaporated, a wax-like layer is formed, which should be left untouched for approximately one hour to crosslink into an elastic silicone rubber film. The silicone glove is initially left on the mold for subsequent assembly steps.

### 2.6. Electrode Preparation

We create sensor electrodes and bundles of conductive traces by casting flat electrically conductive silicone films, unwrapping part shapes from our CAD model, cutting the contours using a cutting plotter (Silhouette Portrait 2) and transferring the patterns onto the glove with stretchable carrier foils.

The part shapes from our CAD model are processed using a modified version of a stretch-minimizing unwrapping tool [5]. In order to manufacture fine-pitched bundles of electrically conductive traces around multi-curved hand shapes, we add support for spacing constraints. Parts and gaps are unwrapped together and automatically separated into their components after unwrapping. Parts and gaps are modeled together as quad meshes in Blender, and each quad is marked as either silicone or spacing through material assignment. We then export the meshes as OBJ files for our unwrapper, process the shapes, and export tool paths as SVG files for the cutting plotter. We control the cutting plotter using the inkscape-silhouette [21].

As transfer foil, we use a TPU film (TPU Film Thermoplatic Polyurethane, Fabimonti, Norderstedt, Germany), thoroughly clean the surface with isopropanol, and apply a zinc stearate release coating using the separating agent dispersion. For the sensor electrodes, we spread a layer of solvent-based electrically conductive silicone directly onto the transfer foil. We apply the film with a doctor blade using strips of plastic tape with a thickness of 140μm on both sides of the carrier film for height reference. The thickness of the silicone film decreases further while drying and curing. For the interconnections, we first spread a conductive silicone film with an uncured thickness of approximately 1mm onto a polyethylene sheet. We then apply a thin layer of silicone paint (Table 1) with a brush for easier identification during assembly. Different colors can be used for different components.

**Figure 5:**
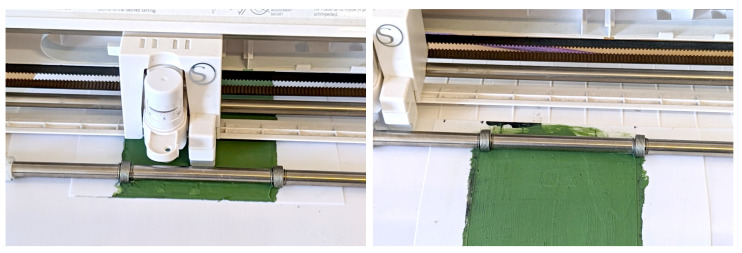
Cutting electrically conductive silicone traces.

**Figure 6:**
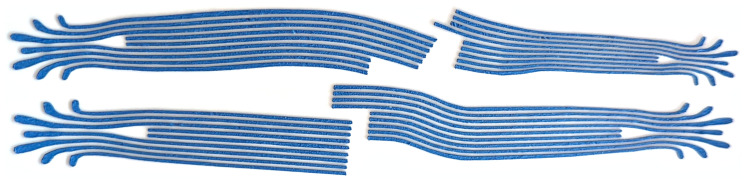
Bundles of electrically conductive silicone traces after cutting, with blue paint for easier identification during assembly.

We feed the carrier foils with the silicone films into a cutting plotter (Silhouette Portrait 2) for shaping the parts. For the sensor electrodes, we set the depth to 2 and the pressure to 5, cutting through both the silicone film and the transfer foil. For the interconnections, we set the depth to 6 and the prssure to 5, only cutting the silicone film. We peel away excess material around the shapes and between the conductive traces. See Figure 5 for the cutting process and Figure 6 for examples of conductive trace bundles.

For the conductive traces, we prepare a second transfer foil with a release coating as before. When casting the cable that connects to the readout module, we cut a narrow strip of thin plastic foil, coat it with a primer (Wacker Primer G790, Nedform BV, Geleen, The Netherlands), and fix it to one end of the carrier foil with a spray-on adhesive (FreshMat, plottiX, St. Gallen, Switzerland) prior to casting the silicone film. The plastic foil later serves as a stiffener to simplify connecting the glove to the readout board and should only cover approximately 1cm at the tip of the silicone cable but not the full length. After preparing the transfer foil, we apply a layer of non-conductive solvent-based silicone dispersion with a thickness of 140μm and wait for the solvent to evaporate and the silicone to cure. We then spread solvent-free non-conductive silicone over the conductive traces, degas in a vacuum chamber, and press the transfer foil with the electrically insulating silicone layer onto the conductive traces, pushing excess liquid silicone out from between the carrier foils. We then let the silicone cure and lift the TPU transfer foil together with the silicone part from the PE sheet.

### 2.7. Dielectric Preparation

We prepare a pressure-sensitive dielectric foil by casting compressible silicone pillars in a CNC-milled mold and covering both sides with thin silicone films. The mold is prepared from a polyoxymethylene (POM) plate by first creating a level surface through face milling and then drilling a set of holes with a radius of 150μm along a hexagonal grid pattern. We spread a layer of solvent-based dielectric silicone onto a polyethylene sheet and let the film dry and cure. The pattern mold is cleaned and covered in solvent-free dielectric silicone (Table 1). The material is degassed in a vacuum chamber, the vaccum is released, and the mold is pressed onto the previously cast silicone film with a mechanical press until the silicone has cured. We peel the carrier foil with the silicone film and dielectric pillars from the mold and wash it with isopropanol to remove separating agent from the silicone surface. We then apply separating agent dispersion to a clean TPU foil (TPU Film Thermoplatic Polyurethane, Fabimonti) and dispense a layer of dielectric solvent-based silicone. We wait for 10 min, place the polyethylene foil with the pillars onto the TPU foil with the dried but uncured silicone and join both layers with a laminator (Laminator Olympia A 230 Plus, Pam’s Naturprodukte Shop, Mellrichstadt, Germany). The dielectric is cut with a cutting plotter according to tool paths generated using our unwrapping tool from the CAD model, similarly to the electrodes (Section 2.6). We set the cutting depth to 5, pressure to 5 and repetition to 2, cutting through the TPU foil and all silicone layers but not through the polyethylene sheet. We then lift the silicone parts together with the pieces of TPU foil from the polyethylene sheet using a knife. The silicone parts should still adhere to the TPU to stabilize the pieces during handling. Silicone surfaces that were touched by human skin should be washed in isopropanol to avoid cure inhibition during assembly.

### 2.8. Assembly Process

We initially keep the glove on the mold and transfer the previously prepared electrode and dielectric parts onto the glove. The target areas are first covered in solvent-based non-conductive silicone as glue. After waiting for 10 min for the solvent to partially evaporate, we place electrode or dielectric elements, with stabilizing TPU transfer foil still attached, on the glove, using the markers on the mold as alignment aids, and press the parts firmly onto the glove and mold for a short moment to ensure good bonding. After waiting 40 min for the adhesive to cure, we peel the transfer foil off and strip the release agent with isopropanol.

We first mount the inner sensor electrodes and then the dielectric elements. Still exposed electrode surfaces are covered in additional solvent-based non-conductive silicone for electrical insulation. We then apply the outer electrodes and finally the interconnecting trace bundles. To further secure the parts and to smoothen the edges, we cover the tip of a toothpick in non-conductive solvent-based silicone and trace exposed part edges.

**Figure 7:**
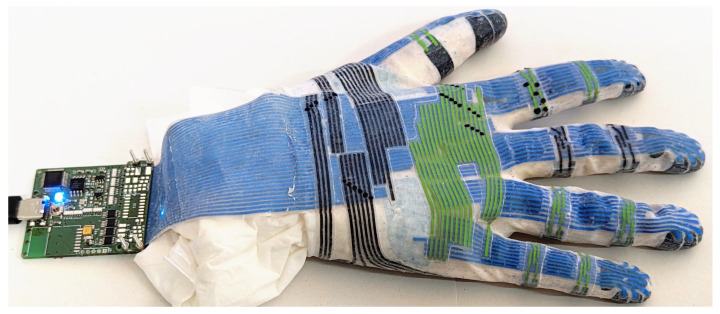
We join conductors on different layers in a partially finished sensor glove by stuffing the glove and punching vertical vias with a conductive silicone-covered needle. Silicone films for a subset of conductors are painted prior to cutting and transfer for easier identification.

**Figure 8:**
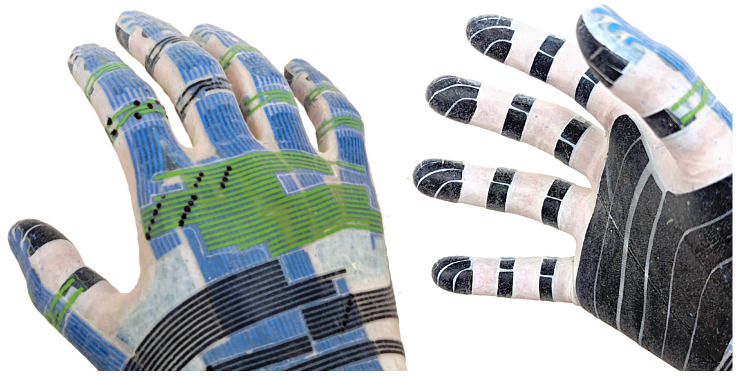
Partially finished sensor glove on a human hand, with sensor elements and conductor bundles transferred to the glove surface. A subset of conductive traces is colored in green and blue for easier identification.

After mounting all components, we create vertical vias to connect corresponding conductive traces and sensor electrodes across different layers, following the previously marked via points in the CAD model. We apply a small amount of Carnauba wax powder as a separating agent to the surface of the glove and remove the glove from the mold, also covering the inside surfaces with powder as they are being exposed. Excess powder is wiped off, and the glove is stuffed with tissue paper. We then clean the outside of the glove with isopropanol, prepare a small amount of solvent-based conductive silicone, dip the tip of a needle into the liquid silicone and repeatedly stick the needle through the glove. Connectivity can be monitored by attaching the glove to the readout board (Section 2.9) or a multimeter. The via stitching process should be repeated until all electrical connections have been established (see Figure 7 and Figure 8). When finished, the vias are covered with drops of non-conductive solvent-based silicone for electrical insulation.

**Figure 9:**
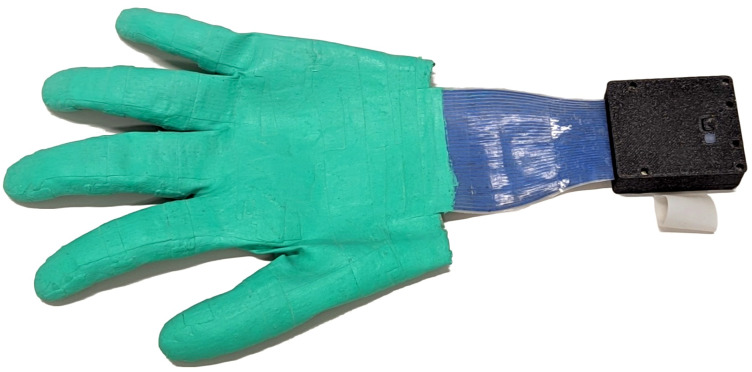
Fully assembled and painted sensor glove with readout module.

The assembly process is completed by painting and post-curing. We remove the tissue paper from the glove, pull the glove over the mold again and wipe the outside surface with isopropanol. Using a brush, we first apply two thin layers of silicone paint and then one layer of transparent solvent-based silicone as varnish. Each layer should be allowed to fully cure for approximately 40 min before applying the next layer. After painting, we apply new Carnauba wax powder, wipe off excess powder and remove the glove from the mold. The glove is baked for one hour at 100 °C to extract potentially remaining volatiles and complete the curing process. The finished glove (Figure 9) can be attached to the readout module (Section 2.9).

### 2.9. Readout Module

**Figure 10:**
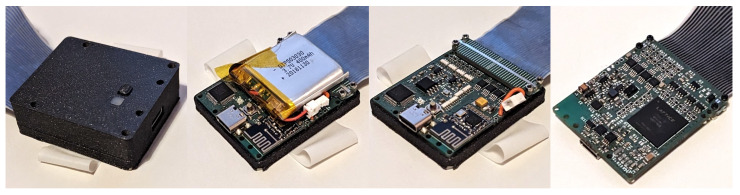
Readout module (**left**), readout board with a battery (**2nd from left**), without a battery and with a visible cable clamp (**2nd from right**), and the lower side of the readout board (**right**).

The sensors are read via a wrist-worn readout module. The readout module (Figure 10) consists of a main circuit board, a clamp for attaching the module to the cable extension of the glove, a plastic case, a wrist band and a battery. The top of the module features a status LED and a control button for powering the device on and off and triggering user-defined actions on a host computer, such as starting and stopping data recording. The back of the module exposes a USB-C port for charging, programming, and debugging. The wrist band is cast from our solvent-based non-conductive silicone formulation. The case is 3D printed on an FFF printer in PLA. The battery is mounted inside the casing above the circuit board. An ECP-5 FPGA (LFE5U-12F-6BG256C, Lattice Semiconductor Corporation, Hillsboro, OR, USA) serves as the central processing unit. The sensor cells are read through delta–sigma conversion via I/O pins on the FPGA and R/C filters [5]. We add additional analog amplifiers (TLV9362, Texas Instruments, Dallas, TX, USA) to increase the excitation voltage to 30V and thereby signal-to-noise ratio. We also add a battery management IC with charging and power path circuitry (ISL9301IRZ, Renesas Electronics, Tokyo, Japan), an IMU (ICM-42605, TDK InvenSense, San Jose, CA, USA), a USB interface IC for programming and debugging (FT232HL, FTDI, Glasgow, Scotland), and a 2.4 GHz radio communication module with an nRF24 transceiver (nRF24L01, Nordic Semiconductor, Trondheim, Norway) and an integrated antenna.

### 2.10. VLSI Design and Verification

We revise a previous VLSI design for tactile sensor readout [5] by developing a more efficient pipelined architecture and adding support for radio communication, data compression, and an IMU. We also implement Verilator [22] test benches with an approximate sensor simulation for design verification. The modules are configured and controlled via a RISC-V softcore [23] through memory-mapped registers.

The tactile readout core generates sine waves with multiple different frequencies as excitation signals, measures input currents at the reception lines, correlates the received currents with sines and cosines of the excitation phases to obtain a complex impedance matrix, and accumulates measurements for transmission. We replace the previous 8-cycle state machine [5], which required 16 cycles for a full complex impedance measurement, with a pipelined parallel architecture that requires only one cycle per complex impedance measurement. A 3-stage sequencer iterates over all sensor cells, obtains the corresponding excitation frequency from a base frequency and a frequency multiplier, and computes the current phase. A two-stage DDS generator reads sine values from a lookup table and optionally attenuates signals according to a gain register. A second DDS generator produces sine and cosine waves for impedance reconstruction. The two-stage impedance reconstruction module correlates the sines and cosines with measurements from an analog-to-digital conversion module and accumulates the results. An additional output stage offers a registered interface for subsequent processing.

The tactile core is embedded together with a data encoder and a memory-mapped tactile matrix in a tactile device module. The tactile core can be exchanged for alternative implementations. The data encoder compresses complex numbers from the tactile correlator as packed 32-bit shared-exponent floating point pairs (similar to [24]) to save bandwidth during transmission. Each packed complex-valued sample consists of two 12-bit mantissae for real and imaginary components and an 8-bit exponent.

In addition to Verilator [22] and Iverilog [25] test cases for data conversion and communication, we develop an approximate simulation model of a capacitive tactile sensor matrix and a corresponding Verilator testbench. The simulation model is intended for fast verification of the VLSI core. We model the excitation drivers as voltage sources with series resistors, the feedback paths of the delta–sigma ADC as RC elements, each sensor cell as a variable capacitor, and assume a fixed ambient capacitance, leading to a simple closed-form solution for the time derivatives, which are numerically integrated. The tactile readout core to be tested is compiled to C++ using Verilator and linked with the simulation model.

To program the FPGA hardware, we use Yosys [26] for logic synthesis, NextPNR [27] for placement and routing, Project Trellis [28] for packing, and ecpprog [29] for flashing.

### 2.11. Recording Setup

We combine our sensor glove with a multicamera system to record multimodal demonstrations for robot learning. Six cameras are mounted on a cuboid metal frame with an edge length of 2 m. Each camera is equipped with a 5 MP image sensor (MT9P001, 1/2.5-Inch 5 Mp CMOS Digital Image Sensor, onsemi, Phoenix, AZ, USA), an ECP5 FPGA (LFE5U-12F-6BG256C, Lattice Semiconductor Corporation), RAM (APS6408L-OBM-BA, AP Memory, Zhubei, Taiwan), an RJ45 connector, and supporting components. The sensor glove is connected via a radio module with an nRF24L01 transceiver. Both the radio module and the cameras connect via bidirectional low-latency LVDS links to an FPGA-based sensor hub. The sensor hub generates synchronization signals, receives and multiplexes sensor data, and connects to a host computer via a USB-3 SuperSpeed connection. The control and driver software on the host computer is implemented as a set of ROS nodes and libraries [30,31].

### 2.12. Multimodal Reconstruction

We produce multimodal reconstructions of human manipulation skills by recording data from the camera system and the sensor glove, reconstructing human hand motions, tracking object features, and mapping motions as well as tactile data to a common articulated 3D hand model.

**Figure 11:**
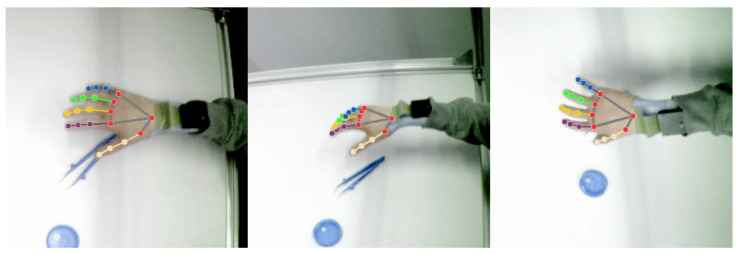
Multi-view false-color images of a sensor glove worn by a human and corresponding landmark detections from a neural hand-tracking network.

We track the 2D pixel locations of 21 human hand landmarks with an existing artificial neural network [32,33]. See Figure 11 for an example. Since the network has been trained on a selection of human hands, we adjust the image colors with a hue filter to match the neural network’s expectations. We combine 2D observations from all cameras to obtain 3D trajectories through trajectory optimization, solving for a sequence of joint angles and 6D wrist poses. We minimize squared reprojection errors as well as joint-space and Cartesian jerk. To avoid local minima, we first initialize the trajectory by purely kinematic reconstruction and introduce dynamic terms in a second optimization phase. As a first initial guess, we fix all joints and only solve for each 6D wrist pose individually using the Kabsch–Umeyama algorithm [34,35]. We then add free variables for the joints and solve for each articulated pose. Finally, we add terms to minimize joint-space and Cartesian-space jerk and optimize the whole trajectory. Six-dimensional wrist poses are represented by quaternions and vectors, and the pose derivatives as six-dimensional vectors. The trajectory reconstruction is implemented in an existing optimization and automatic differentiation framework [36,37,38]. The hand model is exported from MakeHuman [17] and can be adjusted depending on the user.

Tactile data from the sensor glove are projected onto the human hand model according to the same CAD model (Section 2.1) we used for constructing the glove. Measurements are optionally low-pass filtered with a Butterworth filter. The readout matrix is re-arranged according to the spatial arrangement of the sensor cells. It is then projected onto the mesh according to the pre-computed texture coordinates. Intermediate values are estimated through bi-cubic interpolation. The mesh is rendered with OpenGL.

## 3. Results

**Figure 12:**
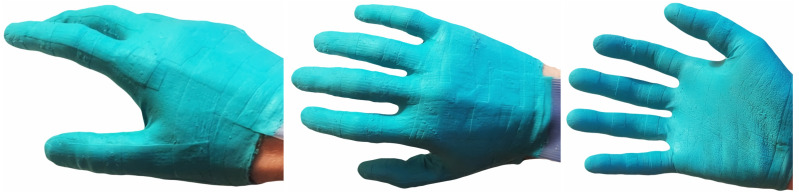
Fully assembled and painted sensor glove on a human hand.

We successfully produced a functioning sensor glove with 102 tactile sensors completely from elastic silicone rubber (see Figure 12). The palm is covered by an irregularly shaped multi-curved 6-by-6 matrix with 30 active cells and each fingertip is covered in a double-curved 3-by-3 sensor matrix. For every distal and proximal phalange, the glove features a three-cell sensor strip. The sensor cells are connected to a wrist-worn readout module via a silicone cable with conductors made from electrically conductive silicone.

**Figure 13:**
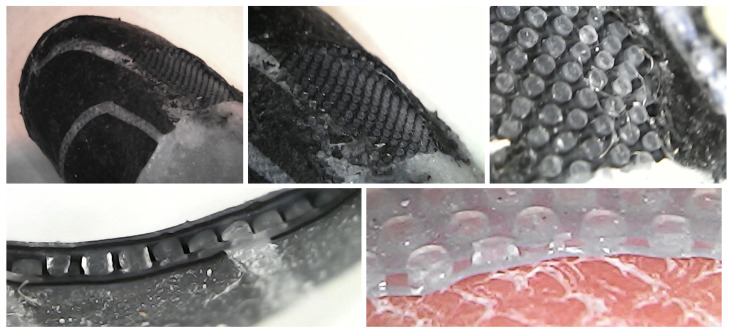
Electrodes (**top left**) and dielectric structure (**top center**, **top right**) on the fingertip of a partially dissected prototype, dielectric pillars between conductive silicone films with insulating gaps between sensor cells (**bottom left**), sensor dielectric pad on a human fingertip (**bottom right**).

Each conductive trace has a width between 500μm and 800μm. Parts of the glove without sensors or conductors, e.g., in the joints, have a thickness of 200μm. Active areas of the glove with tactile sensor cells have a thickness of 400μm (including the base layer and sensors) and the cable from the glove to the readout module has a thickness of 500μm (including conductors and insulation). See Figure 13 for close-ups of partially dissected prototypes. Destructive testing with material samples for dielectric films, silicone conductors on dielectric films, and dielectric pillar structure between dielectric films suggests maximum elongations at break under ideal conditions between 600% and 800%. A bare piece of conductive silicone film breaks when stretched by approximately 600%, while conductive and dielectric films laminated together break when stretched by approximately 700%.

**Figure 14:**
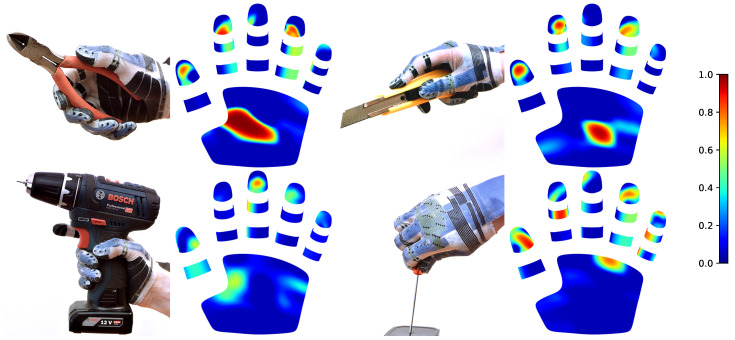
Manipulation experiments with a sensor glove prototype and corresponding tactile sensor readings. Sensor readings are visualized with a colormap (right) and a CAD model of the glove.

Our sensor glove can be used to capture contacts between human hands and objects or tools. The glove is worn on a human hand during several tool-handling scenarios. Tactile measurements are mapped to our CAD model and rendered with bicubic interpolation and an RGB color map. In this first experiment, we use an unpainted version of the glove with visible sensors and conductive traces for illustrative purposes. See Figure 14 for results. For all further experiments, we use the finished painted version.

**Figure 15:**
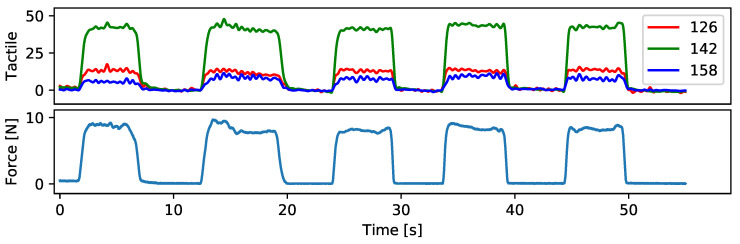
Tactile readings (**top**) from 3 sensor cells located at the tip of the first finger and normal forces (**bottom**) while wearing our sensor glove on a human hand and repeatedly touching a force–torque sensor with the first finger.

Measurements from our sensor glove can be used to estimate mechanical forces. We record normal forces and tactile measurements while a human wears the glove and presses the first finger onto a commercial force–torque sensor (ATI Mini45, ATI Industrial Automation, Apex, NC, USA). Figure 15 shows the recorded normal forces over time and corresponding tactile readings from three adjacent tactile sensor cells. Cell 142 appears to be closest to the contact point. To quantify the results, we compute the Pearson correlation coefficient and find a 99.1% agreement between reference force measurements and tactile readings.

**Figure 16:**
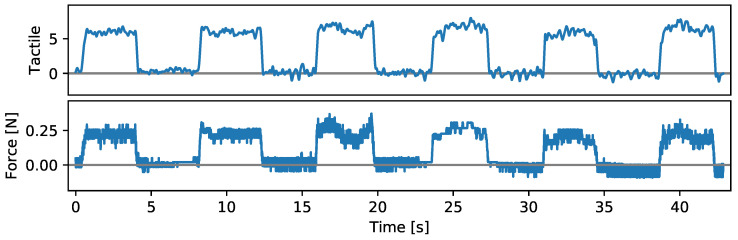
Tactile measurements from our sensor glove (**top**) and force measurements from a commercial force–torque sensor (**bottom**) while a human wears our sensor glove and lightly touches the force–torque sensor with a finger.

Our sensor glove can detect slight touches with low contact forces. Figure 16 shows force outputs from a commercial force–torque sensor (ATI Mini45) and measurements from a single tactile cell on our sensor glove while a human wears the glove and lightly touches the top of the force–torque sensor multiple times with a force of approximately 0.25N. The touches can be felt by the human hand despite the glove. We configure the force–torque sensor for highest sensitivity by selecting the lowest available filter bandwidth of 5Hz via the control panel of the force–torque sensor interface (ATI Net F/T 9105-NETBA) and set the filter bandwidth for our tactile sensor glove to the same value. Both the force–torque sensor and our sensor glove can detect the contacts and show similar noise amplitudes.

**Figure 17:**
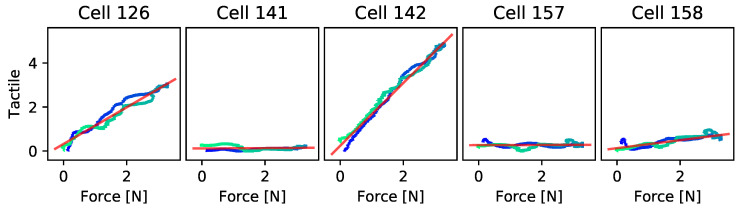
Tactile readings (blue, green) from five different sensor cells at the tip of the first finger of our sensor glove plotted against normal forces, with least-squares linear approximations (red).

The response of our tactile sensor glove to contact forces can be approximately modeled as a linear function. We record tactile and force measurements while a human wearing the sensor glove applies first slowly increasing and then slowly decreasing pressure to a force–torque sensor. For each sensor cell, we fit a least-squares linear approximation. See Figure 17 for plots of the force–tactile response curves and their linear approximations. Cell 142 is closest to the contact point, showing a relative nonlinearity [39] of 0.039.

**Figure 18:**
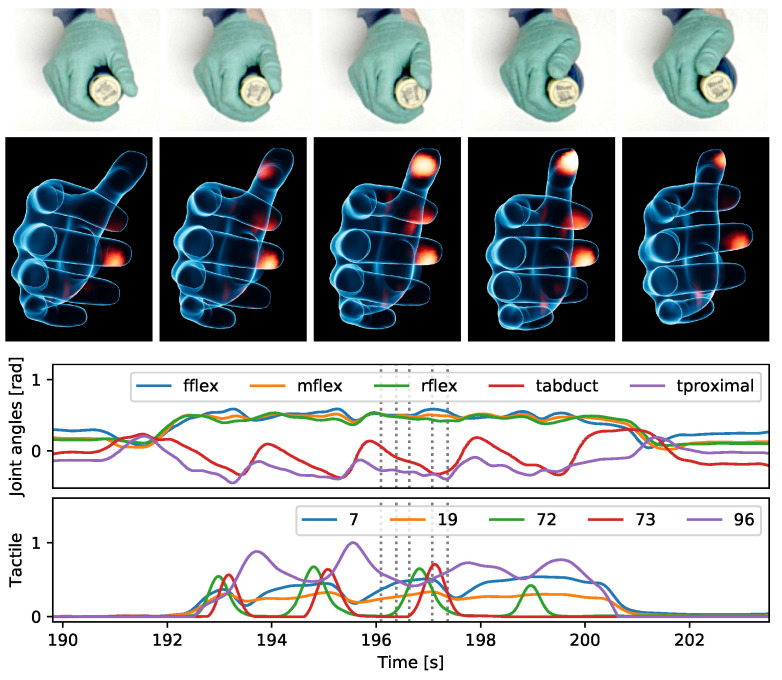
Camera images (**top**), hand pose and contact reconstructions (**Middle**), and joint angle and tactile plots (**bottom**) for turning the lid of a bottle with the thumb. The vertical dotted lines in the plots indicate the time points corresponding to the images and 3D reconstructions shown above.

Our sensor glove can be used together with a multi-camera system to reconstruct dynamic multimodal interactions during dexterous in-hand manipulation. Figure 18 shows 3D reconstructions as well as joint angle and tactile plots for holding a drinking bottle and simultaneously turning the lid with the thumb. The double-curved tactile sensor matrix at the tip of the thumb is able to capture the rolling contact between the lid and the finger. As the finger moves from one side to the other, the contact moves across the fingertip along the opposite direction. The hand motions can still be tracked with existing hand-tracking neural networks [32,33], even while wearing the glove. Depth information can be reconstructed from multiple views through trajectory optimization (Section 2.12). In the example shown in Figure 18, the average fingertip reprojection error of the visual motion reconstruction is 3.52 pixels, corresponding to an average distance between observation rays and reconstructed 3D positions of approximately 3mm.

**Figure 19:**
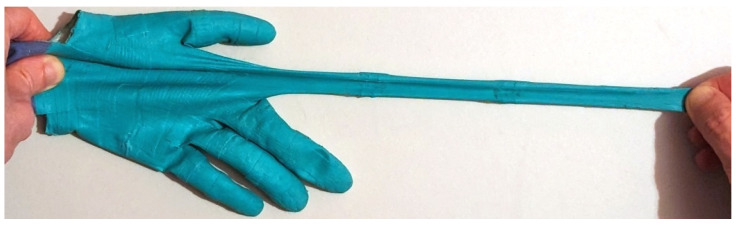
Our sensor glove is completely made from highly elastic rubber-like materials.

The materials of our sensor glove are highly elastic and can adapt to hand geometries, hand poses and flesh deformation under pressure. Figure 19 shows one of the fingers being stretched to multiple times its original length, after which the glove returns to its original shape (Figure 12) and remains functional. Our glove prototype has been successfully used over four months without damage or noticeable material degradation.

## 4. Discussion

We can produce thin and elastic tactile sensor gloves completely from rubber-like stretchable materials. These gloves can act as artificial second skins to capture tactile information, which can be mapped to force estimates. Together with 3D motion tracking, we can produce multimodal reconstructions of human dexterous manipulation skills. As our main intended application, we expect such data to be useful for teaching manipulation tasks to robots. Experimental results suggest that our sensor glove reaches a similar normal force discrimination ability as human hands [40,41]. The thickness of our sensor glove is similar to that of the epidermis (top layer of the skin) on human fingers [42]. Our sensors are intrinsically multi-curved to match the shape of human hands and can additionally stretch around joints and with deformations of human flesh under pressure. While systematic bio-compatibility evaluation and long-term user studies could be a topic for future work, we expect our glove to be safe for human use. Both the conductive and the non-conductive parts are manufactured from addition-cure silicone rubber. The conductors are produced from silicone rubber with a special food contact-grade carbon black. This allows us to forego brittle inelastic materials [11,43,44], hazardous or corrosive conductive liquids [45,46], as well as new and experimental particulate fillers with unclear health and environmental risks [47,48,49]. Our conductive materials are nevertheless more stretchable than those used in previous and related works [5,48,50]. Instead of traditional wires, we can also produce the cables with stretchable silicone conductors. The readout module is placed on the wrist to not impede dexterity and can successfully read the sensors across the silicone flat cables. We have successfully used a first prototype for months without damage to the glove or adverse effects to humans. While for our own work in teaching by demonstration, we are currently most interested in capturing normal forces, an interesting direction for future and related work could be additional shear force measurement. We also considered integrating multimodal and marker-based motion tracking [51,52,53] but found that state-of-the-art neural networks for markerless hand tracking can already detect our glove sufficiently well for our current applications. Markerless tracking might also facilitate integration with existing visual-only datasets and the training of multi-modal foundation models from diverse data sources.

## Figures and Tables

**Table 1:** Material formulations for producing elastic tactile sensor gloves.

Precursor Chemical	Conductive Film	Dielectric Film	Pigmented Film	Dielectric Casting
Hexamethyldisiloxane (CAS 107-46-0, WACKER AK 0,65, silikonfabrik.de)	400g	400g	200g	−
Vinyl-functional siloxane gum (CAS 67762-94-1, Vinyl Gum 0.074, Nedform BV)	53g	80g	20g	−
Vinyl-terminated PDMS 2000cSt (CAS 68083-19-2, Vinyl Dimethicone 2.000 cSt, Nedform BV)	−	−	−	400g
Methylhydrosiloxane-DMS copolymer (CAS 69013-23-6, Crosslinker 200, Evonik Industries AG)	632mg	673mg	198mg	1.765g
Hydride-terminated PDMS (Modifier 2.6, both sides Si-H end-capped, Nedform BV)	−	−	−	11.674g
Hydroxy-terminated PDMS (CAS 70131-67-8, Polydimethylsiloxane, hydroxy terminated, M.W. 4200, VWR International GmbH)	4g	−	490mg	−
Hydrophobic fumed silica (CAS 68909-20-6, Aerosil R 812 S, Evonik Industries AG)	−	16g	−	34g
Carbon black (CAS 1333-86-4, Food Contact Royale Black PP802, PCBL Ltd., Profiltra)	40g	−	−	−
Titanium dioxide	−	−	6g	−
Pigments (Phthalocyanine (PG7, PG36, PB), Ultramarine Blue, Chromium Oxide Green - Kremer Pigmente)	−	−	2g	−
Zinc oxide	−	800mg	−	800mg
Zinc stearate	−	−	50mg	800mg

## Data Availability

Source code, design files and sample data are available at https://github.com/TAMS-Group/tams_glove.

## Abbreviations

The following abbreviations are used in this manuscript:

ADC	Analog-to-digital converter
CAD	Computer-aided design
CAS	Chemical Abstracts Service
CNC	Computer numerical control
DDS	Direct digital synthesis
DMS	Dimethylsiloxane
FFF	Fused filament fabrication
FPGA	Field programmable gate array
IC	Integrated circuit
IMU	Inertial measurement unit
LED	Light-emitting diode
LVDS	Low-voltage differential signaling
OBJ	Wavefront object file
PDMS	Polydimethylsiloxane
PE	Polyethylene
PLA	Polylactic acid
POM	Polyoxymethylene
RAM	Random-access memory
RC	Resistor–capacitor
RGB	Red, green and blue
RISC	Reduced Instruction Set Computer
ROS	Robot Operating System
SLA	Stereolithography
SVG	Scalable Vector Graphics
TPU	Thermoplastic polyurethane
USB	Universal Serial Bus
VLSI	Very large scale integration
